# Association of Celiac Disease With Pembrolizumab

**DOI:** 10.7759/cureus.15565

**Published:** 2021-06-10

**Authors:** Ashish Sethi, Alexander Helfand, Lame Balikani, Mark Bunker, Gene Finley

**Affiliations:** 1 Medical Oncology, Allegheny Health Network, Pittsburgh, USA; 2 Hematology & Oncology, Allegheny Health Network, Pittsburgh, USA; 3 Pathology, Allegheny Health Network, Pittsburgh, USA

**Keywords:** nivolumab, pembrolizumab, celiac disease, immune-related adverse effects, ipilimumab, immune check point inhibitor colitis, immune check point inhibitor induced celiac disease, gluten-free diet, cancer immunotherapy, immune checkpoint inhibitors

## Abstract

Immune checkpoint inhibitors (ICIs) in the recent times have transformed the landscape of the management of many solid tumors. Unfortunately, many immune-related adverse effects are associated with ICIs, which lead to a negative outcome in cancer treatment. We present a case of a 63-year-old female with metastatic adenocarcinoma of unknown origin, who developed celiac disease during the course of treatment with pembrolizumab. Association of celiac disease with this form of immunotherapy has never been documented before.

## Introduction

Immunotherapy has acquired a significant role in the management of many solid tumors especially non-small cell lung cancers (NSCLCs) and melanoma. Pembrolizumab, an immune checkpoint inhibitor (ICI), is a humanized monoclonal antibody directed against programmed death receptor 1(PD-1), which blocks the negative immune regulatory signals of the PD-1 receptor manifested by T cells, B cells, or macrophages [1]. However, as pembrolizumab blocks the immune system checkpoints, it can cause T cells to attack healthy cells, leading to different autoimmune diseases, which are termed as immune-related adverse events (irAEs). The most common irAEs observed are pneumonitis, colitis, hepatitis, hypophysitis, thyroiditis, and nephritis [2].

We report an unusual presentation of possible association of celiac disease with pembrolizumab, which is also an autoimmune disease. To the best of our knowledge, the occurrence of celiac disease in a patient after pembrolizumab therapy has been rarely documented in the medical literature.

## Case presentation

A 63-year-old Caucasian female was diagnosed with metastatic adenocarcinoma of unknown primary origin. Computed tomography (CT) scan findings showed mediastinal lymphadenopathy and left hilar adenopathy. Physical examination was positive for left supraclavicular lymphadenopathy. Her medical history included hypertension, hypothyroidism, and left-sided high-grade ductal carcinoma of the breast. She reported smoking cigarettes (one pack per day) for the past 40 years and denied alcohol consumption or other illicit substance use.

Needle core biopsy performed on the left supraclavicular lymph node demonstrated metastatic adenocarcinoma favoring breast as the primary source of origin. The tumor cells from biopsy results were positive for CK7 and E-Cadherin and focally for GATA3 and CDX-2, and were negative for CK5/6, CK20, TTF-1, p40, napsin-A, ER, PR, HER2, mammaglobin, and GCDFP-15 immunostains. Further molecular profiling did not reveal atypical driver mutation, but she did have KRAS G12C, STK-11, and a PALB2 mutation, which is considered as germline mutation.

Based on her evaluation and clinical assessment, she was treated with a combination of carboplatin, paclitaxel, and pembrolizumab. After five cycles of therapy, treatment was held because of immune-mediated grade 3 colitis that was unresponsive to steroids. The patient presented with complaints of diarrhea without blood, mild diffuse abdomen pain, and weight loss of 10-12 pounds since few months after starting pembrolizumab. Upon evaluation for infliximab for her unresponsive grade 3 colitis, she was found to have a positive QuantiFERON gold test. Hence, prophylactic anti-tubercular treatment with isoniazid (INH) for nine months was started in view of her latent tuberculosis (TB).

In the interim, the patient underwent colonoscopy, which did not reveal any evidence for colitis as she had complaints of persistent chronic diarrhea that was associated with steatorrhea. Later, the patient also developed INH-induced hepatitis, and INH was also withdrawn from her treatment plan for latent TB. Subsequently, drug-induced hepatitis was resolved.

**Figure 1:**
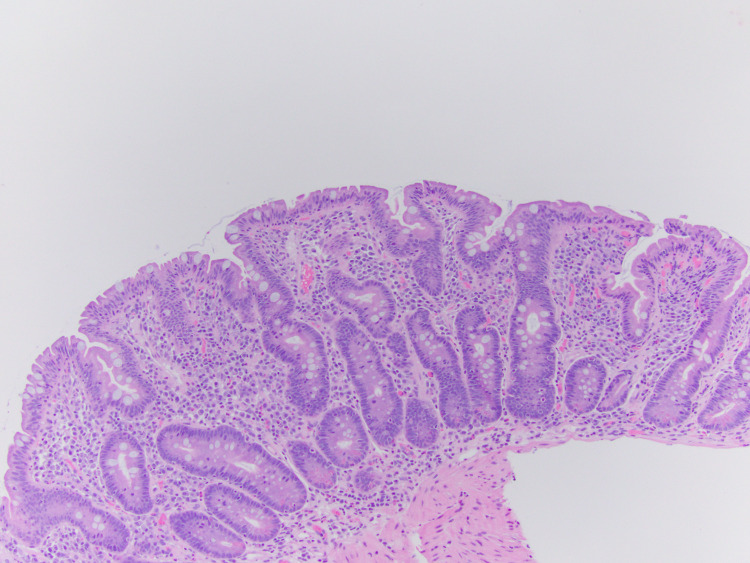
Villous blunting crypt hyperplasia (10x)

**Figure 2:**
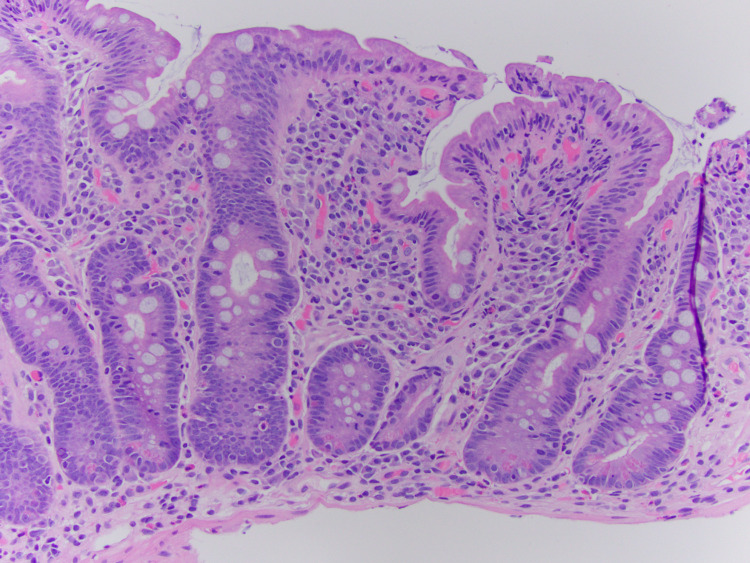
Villous blunting crypt hyperplasia (20x)

**Figure 3:**
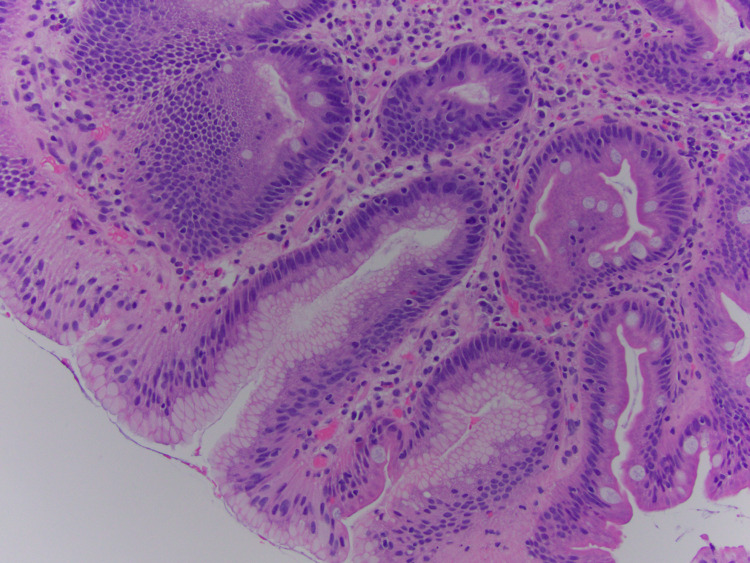
Gastric fovelolar metaplasia

Upper gastrointestinal endoscopy with biopsy was then performed due to refractory diarrhea, which was conclusive of evidence for celiac disease (Figures 1-3).

Also, important serological tests performed supported the endoscopic findings of celiac disease (Table 1). The patient was managed with strict gluten-free diet after which her diarrhea was resolved.

**Table 1:** Lab values IG, immunoglobulin; HBsAg, hepatitis B surface antigen; HCV, hepatitis C virus; WBC, white blood cell

Labs	Results
Anti-transglutaminase level, IgA	<2
Endomysial antibody, IgA	Negative
Anti-transglutaminase, IgG	5 U/mL
Anti-gliadin IgA	49 U/mL
Anti-gliadin IgG	41 U/mL
HBsAg	Non-reactive
HCV antibody	Non-reactive
Hemoglobin	13.1 g/dL
WBC	8,000 k/mcl

## Discussion

Many ICIs have been noted to increase overall survival and progression-free survival in different malignancies. Some of the approved immunotherapies currently in use are given in Table 2.

**Table 2:** Immune checkpoint inhibitors CTLA-4, cytotoxic T-lymphocyte-associated protein 4; HCC, hepatocellular carcinoma; MSI, microsatellite instability; NSCLC, non-small cell lung cancer; PD-1, programmed death receptor 1; PD-L1, programmed death-ligand 1; RCC, renal cell carcinoma

Immunotherapy	Target receptor	Indication
Pembrolizumab	PD-1	Melanoma, NSCLC, squamous cell carcinoma of the head and neck, urothelial carcinoma, gastric cancer, classic Hodgkin’s lymphoma
Nivolumab	PD-1	Melanoma, NSCLC, RCC, HCC, squamous cell carcinoma of the head and neck, urothelial carcinoma, colorectal cancer with high MSI, classic Hodgkin’s lymphoma
Atezolizumab	PD-L1	NSCLC, urothelial carcinoma
Avelumab	PD-L1	Merkel cell carcinoma, urothelial carcinoma
Durvalumab	PD-L1	Urothelial carcinoma
Ipilimumab	CTLA-4	Melanoma

irAEs usually develop after few weeks into the treatment with any immunotherapy. However, it may even occur after the cessation of ICIs. Many studies have indicated that with anti-CTLA-4 and anti-PD-1 agents, dermatologic toxicity is seen as an early manifestation [3,4]. As anti-PD-1 or anti-PD-L1 (programmed death-ligand 1) agents are sometimes given for months to years, most studies indicate that prolonged treatment does not result in an increased cumulative incidence of irAEs [5].

Celiac disease is an autoimmune disorder characterized by small intestinal enteropathy that may be diagnosed at any age and affects many organ systems. The classic presentation of celiac disease is diarrhea, which may be accompanied by abdominal pain. However, diarrhea as the main presenting symptom has been noted in less than 50% of diagnosed cases in the past [6]. Some silent clinical features in celiac disease include iron deficiency anemia, osteoporosis, or incidental recognition at endoscopy performed for other reasons such as gastroesophageal reflux disease [7]. Infrequent clinical signs or symptoms constitute abdominal pain, constipation, weight loss, neurologic symptoms, dermatitis herpetiformis, hypoproteinemia, hypocalcemia, and increased liver enzyme levels [8]. The pathogenesis of celiac disease involves an external trigger (gluten), change in intestinal permeability, human leukocyte antigen (HLA) recognition, and innate and adaptive immune responses to gluten peptides recognizing as self-antigens (e.g., transglutaminase), leading to celiac enteropathy [9,10].

Serum immunoglobulin (Ig) A and IgG anti-tissue transglutaminase antibodies in persons with IgA deficiency are recommended for initial serological testing in celiac disease. IgA antiendomysial antibody level is 100% specific for active celiac disease [11]. Measurement of deamidated gliadin peptide antibodies of the IgG class has also been introduced as an alternative test and is reported to have better sensitivity and specificity compared to levels of IgG anti-tissue transglutaminase antibodies as a screening tool for celiac disease in IgA deficient individuals [12].

Gluten-free diet is the main treatment for celiac disease, which keeps the disease under remission. Dietary nonadherence to gluten-free diet has been the cause of persistent symptoms of diarrhea and abdominal pain in many cases. Long-term sequelaes of untreated celiac disease can lead to small intestinal adenocarcinoma, refractory sprue, and enteropathy-associated T-cell lymphoma. The response to a gluten-free diet is variable. Approximately 70% of patients show clinical improvement within two weeks [13].

Badran et al. in their study identified patients who developed ICI-induced celiac disease [14]. Among the patients who developed celiac disease with the ICIs, two had NSCLC, one had extraskeletal myxoid chondrosarcoma, and one had squamous cell carcinoma of the tonsil [14]. Furthermore, the responsiveness of ICI-induced celiac disease to gluten-free diet alone in five (62%) out of the eight cases illustrated that gluten is an important antigen driving ICI-induced celiac disease, similar to standard celiac disease [14]. Also, Gentile et al. observed ipilimumab-associated celiac disease in a patient with castration-resistant metastatic prostate cancer, who presented with refractory diarrhea after three doses of this or of immunotherapy [15].

A gluten-free diet is a reasonable treatment strategy for patients with ICI-induced celiac disease. Thus, tailoring proper therapy can avoid systemic immune suppression preventing unnecessary discontinuation of ICI treatment. Thus, suspecting ICI-induced celiac disease early in presentation can have substantial clinical implications for many physicians. Esophagogastroduodenoscopy alongside a flexible sigmoidoscopy or colonoscopy for the evaluation of immunotherapy-induced colitis should be included as a part of normal routine evaluation.

## Conclusions

ICI-induced celiac disease resembles ICI-induced colitis clinically and histologically but shares the serological features and response to gluten-free diet with the classic celiac disease. We conclude that celiac disease due to immunotherapies is biologically similar to ICI-induced colitis and is likely a variant of ICI-induced colitis or duodenitis. However, the treatment strategies in both forms of entities differ, with ICI-induced celiac disease often improving with gluten-free diet alone, whereas ICI-induced colitis or duodenitis needs systemic immunosuppression.
